# The Effect of Healing Gardens among Care Residents with and Without Dementia in Nigeria

**DOI:** 10.1002/alz.085162

**Published:** 2025-01-09

**Authors:** Funmi Akindejoye, Uchenna Ezedinma, Susanne Rorh

**Affiliations:** ^1^ Global Brain Health Institute, TCD, Dublin Ireland; ^2^ University of Tasmania, Tasmania, TAS Australia; ^3^ University of the Sunshine Coast, Australia, Sunshine coast, QLD Australia; ^4^ Global Brain Health Intitute, TCD, Dublin, Dublin Ireland; ^5^ Massey University, University of New Zealand, New Zealand, New Zealand New Zealand

## Abstract

**Background:**

A healing garden is a non‐pharmacological approach that supports the interaction of humans and elements of nature towards improving well‐being and quality of life. It has gained increased utilization in managing the health outcomes of older persons with and without dementia. However, there is a paucity of evidence of its use and benefits in African countries with increasing prevalence of dementia, such as Nigeria. This study aims to co‐design a low‐maintenance healing garden intervention and measure its impact on the psychosocial measures and quality of life of participating residents and caregivers, from two care home facilities in Lagos state, Nigeria.

**Method:**

The study co‐designed and developed a healing garden in two selected care facilities and employed validated tools to measure the quality of life, agitation, and depression at baseline and three months after the intervention. Also, surveys were used to elicit further information from caregivers, three months after the co‐design, and development of individual healing gardens.

**Results:**

The study revealed that the low‐maintenance healing garden improved the quality of life and experiences of agitation but no significant change in depression measures among residents with and without dementia. Further, caregivers reported a positive benefit of the healing garden on their work‐life experience and the residents’ well‐being.

**Conclusion:**

Evidence from this study on the benefits of healing gardens to older persons with or without dementia in Nigeria is preliminary. Future research using a more multidimensional and broad research design is warranted.

